# The Bacterial Treatment of Sewage

**Published:** 1899-10-21

**Authors:** 


### THE BACTERIAL TREATMENT OF SEWAGE.

The sewage experiments which have been carried on for some time back at Crossness by Dr. Clowes and Dr. Houston, on behalf of the County Council, strongly confirm the opinion that the bacterial treatment of sewage affords the true solution of the difficulties which have so long stood in the way of the universal adoption of the water carriage method of conservancy. Chemical treatment is admitted to be but a makeshift at the best, while irrigation, which at present holds the field, involves the use of very large areas of land, which in some cases cannot possibly be found within reasonable distance of the towns to be drained. Bacterial treatment, on the other hand, requires an amount of land which may be fairly called very small in comparison with what would be wanted for irrigation, while it produces a far better effluent than is given by chemical treatment, and at the same time gets rid of the sludge difficulty. We do not gather from the report what is the manurial value of the effluent; but we should imagine that it would be as great as, if not greater than, that of the sewage itself. It must not, then, be imagined that the adoption of the process will of necessity detract from the value of such irrigation farms as already exist, for it seems not impossible that the addition of bacterial filter beds may remove many of the difficulties which have stood in the way of the successful management of such farms. A given quantity of land will evidently absorb and utilise a good deal more sewage when the prutrescible matters are in solution than when they are in the solid form, while the tendency to produce a nuisance will be far less. In regard to this side of the question a paper read by Mr. Scott-Moncrieff at the recent Congress of the Sanitary Institute is of considerable interest, for he showed that by aid of the bacterial process not only is purification of the sewage obtained, but that a supply of nitrogen ready prepared for plant life is provided. He said that at the present moment disappointment caused by the broken promises of the past, in regard to the profit to be derived from sewage farms, seemed to make everyone look upon sewage as a nuisance to be got rid of; but now that by the bacterial method the proper adjustment of the sewage, in chemical constitution, to the requirements of plant life had been attained, profit to the community would surely follow the use of such highly nitrified effluents for irrigating purposes.

